# Cases from Surgical Practice

**Published:** 1888-03

**Authors:** Hunter P. Cooper

**Affiliations:** Atlanta, Ga., Professor of General and Medical Chemistry, Atlanta Medical College


### CASES FROM SURGICAL PRACTICE.

---

BY HUNTER P. COOPER, M. D., ATLANTA, GA.,

Professor of General and Medical Chemistry, Atlanta Medical College.

---

CASE I.—REMOVAL OF CYSTIC TUMOR OF LABIUM MAJUS—PROFUSE HEMORRHAGE FROM RUPTURE OF A LARGE VEIN AFTER OPERATION.

N. N., aged 33, a colored woman of unusual intelligence, applied to me in July, 1886, for relief from a growth which was blocking up the entrance to the vagina. Examination revealed a tense elastic tumor the size of a guinea egg, situated in the left labium majus. The diagnosis of cystic tumor was easily reached.

The history she gave was that it had been slowly growing for four years, giving pain only during menstruation and sexual intercourse. The operation for its removal was performed September 23, Dr. N. O. Harris kindly assisting me. The labium was shaved then thoroughly washed with soap and water, and finally with bichloride of mercury solution (1 to 2,000). Hypodermic injections of a 10% solution of cocaine were made around and above the tumor in five different places. An incision about two inches long was then made on the outer side of the labium and the tumor carefully dissected out without cutting into it. No pain was experienced during the operation, and no vessel was cut that required a ligature. The wound was thoroughly irrigated with the bichloride solution, closed with cat-gut sutures and dressed antiseptically.

An hour after I left the house, I was hastily summoned by the unpleasant statement that the patient was bleeding to death. I found the dressings and bedding saturated with blood, and on removing the former the blood was seen rapidly flowing from the wound in the intervals between the stitches. The labium was so distended by clotted blood that it had reached the size of a large orange. I immediately cut all the stitches, turned out the clots and tried to find the bleeding vessel; but a few moments' search satisfied me of the futility of attempting to apply a ligature. I was convinced that the hemorrhage was venous, and that the vein from which it proceeded was very large. In a very short time the patient had lost a large quantity of blood, was gasping for breath, and complaining of everything turning black before her eyes. I had no styptic with me, and no assistant save an old negro woman. I directed her to bring me a kettle of hot water, and soaking sponges in this stuffed them into the wound. I then made pressure against the parts with my hand, thus restraining further hemorrhage, while the old woman hurried to the nearest drug store for tincture of the chloride of iron. During her absence the patient had several attacks of syncope. When my messenger returned I saturated a sponge in the ferric chloride and packed it into the wound; over this I placed a firm compress which I retained in place with a T bandage. I had meanwhile sent for Dr. Harris to come to my assistance; when he arrived the bleeding was completely checked, and we could give all our attention to reviving the patient. She was suffering extremely from the loss of blood, the heart's action was very feeble, the respiration, gasping and sighing, great restlessness, thirst and mental anxiety. I tried whisky, morphine and digitalis both by the stomach and hypodermically with but little success. At the suggestion of Dr. Harris I tried hypodermic injections of atropia combined with a small amount of strychnine, and soon had the satisfaction of seeing the heart's action become slower and stronger, and the patient rallied without any more trouble. The subsequent history of the case possesses no special interest; the wound rapidly healed after the removal of the sponge.

The hemorrhage was undoubtedly due to a rupture of one of the large veins in the *bulbus vestibuli*, whose wall had been weakened by the removal of the tumor; possibly I had nicked the vein with the point of my knife. Its rupture was produced by her moving about in bed after I left the house.

CASE II.—STRICTURE OF THE URETHRA COMPLICATED BY PERINEAL ABSCESS—EXTERNAL URETHROTOMY—RECOVERY.

W. K., aged 27, married, came to me from an adjoining State in July, 1887, with the following history:

In November, 1886, he contracted a severe gonorrhœa, his first and only attack. In the following January the size of the stream of urine began to diminish and a swelling appeared in the perineum. This perineal swelling was hard, very painful (confining him to bed for three months) and slow in its development. At the end of three months it was opened in the median line and a large amount of pus evacuated. During micturition a considerable proportion of urine came through the perineal incision, thus demonstrating the character of the abscess. After this his condition improved a good deal; the perineal opening gradually contracted and finally closed, and he was able to attend to his business again. Some of the symptoms, however, remained, viz., painful and frequent micturition and diminution in the size of the stream passed.

Early in July a friend playfully gave him a hard slap between the buttocks as he was leaning over a counter. Severe pain at once resulted and the whole perineum rapidly swelled. A purulent discharge from the urethra appeared and the pain in urinating became more marked. After suffering greatly for nearly a week, he came to Atlanta for treatment.

*July 12th.*—PRESENT CONDITION.—Examination shows a hard red swelling on right side of perineum, almost far enough back to be mistaken for ischio-rectal phlegmon. This swelling is very painful and tender. The meatus is greatly contracted, red and discharges muco-pus. Micturition is both frequent and painful, and there is great obstruction to the flow of the urine.

*July 15th.*—Hot poultices have been kept to the perineum for three days, and now there is marked fluctuation in the swelling.

*Operation.*—Present, Drs. Elkin, Harris and Greene. The meatus was first cut to 36 Fr. The perineal abscess was next incised and about one ounce of pus evacuated. Sweet oil injected into the penis came out through the abscess cavity, thus proving a perforation of the urethral wall. Gouley's tunneled sound was then passed into the urethra until it was stopped deep down in the perineum; the urethra was opened in the perineum on the groove of the sound, the canal drawn well into sight by silk loops passed through the cut edges, and a tight, dense stricture was then cut in the membranous portion of the urethra. No. 36 Fr. was then passed into the bladder. The hemorrhage was quite profuse during the operation, but gradually ceased, and the patient was put to bed in good condition.

*July 16th, 10 a. m.*—Patient has had three severe urethral chills since the operation, one of them occurring immediately after he passed his urine. I drew off nearly a pint of urine with a soft catheter, and washed out the bladder with a tepid solution of borax. Temperature 102° F. in axilla.

*July 17th.*—The temperature rose yesterday until by noon it had reached 103½. Antipyrine in 15-grain doses reduced it nearly to normal and caused very profuse sweating. Temperature is now 99½° F

After this date the patient did very well; sounds were passed every second or third day until July 29th, when an acute epididymitis occurred on the left side. This gradually subsided under the usual treatment, and he improved very rapidly; the perineal wound is closing, the larger part of the urine passing through the penis.

*August 15th.*—To-day the patient has a temperature of 104° in axilla, and very marked febrile symptoms with vague pains in the right testicle and groin.

*August 16th.*—Very marked epididymitis has developed on the right side.

*September 1st.*—The two attacks of epididymitis caused me to stop passing instruments for over three weeks; during this time I find a stricture has formed at the peno-scrotal junction. Dr. Elkin saw the patient in consultation with me yesterday, and advised internal urethrotomy and enlarging the meatus. This was done with his assistance, and No. 34 Fr. was passed to the neck of the bladder.

*September 10th.*—No 34 Fr. was passed yesterday with ease. Patient recovered rapidly from the last operation and left for home last night.

Several months after he left, I heard from a friend of his that he was well apparently and was actively engaged in his profession.

The case possesses a good many interesting points—an especial one, in the fact that antipyrine completely controlled the urethral fever.

CASE III.—CONTRACTED MEATUS URINARIUS ATTENDED BY MARKED SYMPTOMS OF CALCULUS—PERFECT CURE BY ENLARGING MEATUS.

J. D., a colored janitor, aged 28, suffered for several years with the following symptoms: Great obstruction to the flow of urine, the stream being small and crooked. Micturition is frequent both during the day and at night, is painful and is accompanied by much straining. At times the stream is cut short before the bladder is emptied. Occasionally there is considerable blood in the urine.

In addition to these symptoms, he says that when a child he often passed small calculi through the penis, their passage causing him to scream with pain.

Examination shows a meatus which is so contracted that it will admit no larger instrument than No. 8 Fr.

*December 20th, 1886.*—The meatus was cut to 34 Fr., Drs. Elkin, Harris and Greene being present. This was done as a preliminary step to exploring the bladder for a calculus. Unavoidable circumstances caused so much delay that it was decided to postpone the exploration.

A stricture of large calibre was found two inches from the meatus; another at the bulbo-membranous junction, which we took to be spasmodic.

After the healing of the meatus, the symptoms had so greatly improved that I no longer suspected vesical calculus. For two months I passed a full-sized instrument into the bladder twice a week, at the end of which time all his symptoms had disappeared entirely, and his urine passed from him in a full, bold stream without pain or straining.

I saw him quite often for several months thereafter and he remained perfectly well.

